# Differentiation of functional astrocytes from human-induced pluripotent stem cells in chemically defined media

**DOI:** 10.1016/j.xpro.2021.100902

**Published:** 2021-10-20

**Authors:** Sylvain Perriot, Mathieu Canales, Amandine Mathias, Renaud Du Pasquier

**Affiliations:** 1 Laboratory of Neuroimmunology, Neuroscience Research Centre, Department of Clinical Neurosciences, Lausanne University Hospital (CHUV) and University of Lausanne, 1015 Lausanne, Switzerland; 2 Service of Neurology, Department of Clinical Neurosciences, Lausanne University Hospital (CHUV) and University of Lausanne, 1015 Lausanne, Switzerland

**Keywords:** Cell Biology, Cell culture, Neuroscience, Stem Cells, Cell Differentiation

## Abstract

This protocol describes how to obtain human astrocytes from human-induced pluripotent stem cells (hiPSCs) in chemically defined media, without the use of fetal bovine serum (FBS). FBS eases the differentiation of astrocytes but also deeply alters their phenotype, as compared with their *in vivo* counterparts. Our protocol generates hiPSC-derived astrocytes displaying a phenotype and functions similar to human primary astrocytes, including adequate response to inflammation, neurotransmitter uptake, and trophic support to neurons.

For complete details on the use and execution of this protocol, please refer to Perriot et al. (2018).

## Highlights

• Differentiation of astrocytes from hiPSCs in chemically defined media
• Generation of highly characterized resting human astrocytes
• Full functionality of hiPSC-derived astrocytes as compared with primary cells
• Suitable model to study human astrocyte responses to inflammation

## Graphical abstract

**Figure:**
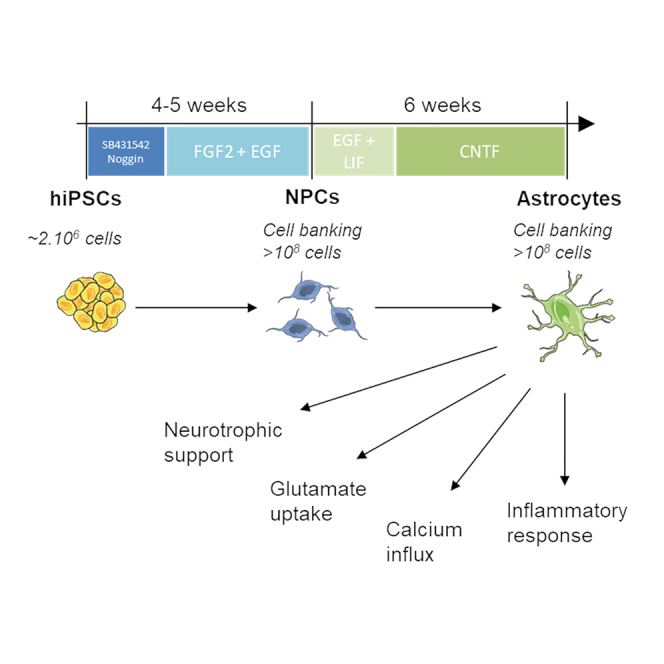

## Before you begin

The execution of this protocol requires knowledge of pluripotent stem cell culture techniques, which are not covered here. Human iPSCs used to develop this protocol have been cultured in StemMACS human iPSC Brew medium (Miltenyi) or TeSR-E8 medium (Stemcell Technologies). We do not foresee any limitation using other culture media for hiPSC culture but did not assess it experimentally. Human iPSCs and differentiated cells were cultured in a humidified 37°C incubator and 5% CO_2_.

For optimal results, the protocol should be initiated using hiPSCs at 70%–80% confluence without any differentiated cells. The protocol was successfully carried out using hiPSCs from passage 10 to 40.

Prior to beginning the differentiation protocol, prepare media, solutions, and matrix-coated culture dishes. Once prepared, media supplemented with N-2 and B-27 supplements can be kept at 4°C for 2 weeks. However, other supplements (growth factors and others) should be added freshly to the medium every time of use. All media should be brought to room temperature (20°C–24°C) or 37°C prior to adding on the cells.

### Poly-L-ornithine coating

1. Dilute poly-L-ornithine at 1:5 in sterile water and add it to the dish in order to cover the entire surface. Incubate at room temperature for 2 h.
2. Remove poly-L-ornithine solution and add laminin (2 μg/mL diluted in sterile water) in order to cover the entire surface. Incubate at room temperature for 2 h. Coated dishes can be kept in this solution for 2 days at 4°C before use.

### Matrigel coating

3. Thaw matrigel at 4°C.
4. Dilute matrigel in cold DMEM/F12 as per dilution factor mentioned on the datasheet (final concentration of 100 μg/mL).
5. Add the diluted matrigel to the dish in order to cover the entire surface.
6. Incubate at room temperature for 60 min. Coated dishes can be kept as such at 37°C for 3 days (no wash needed before use).

## Key resources table

**Table:** 

REAGENT or RESOURCE	SOURCE	IDENTIFIER
**Antibodies**		

anti-GFAP rabbit IgG (dilution 1:200)	Sigma-Aldrich	Cat#AB5804
anti-S100β rabbit IgG (dilution 1:200)	Abcam	Cat#AB52642
anti-GLAST rabbit IgG (dilution 1:200)	Abcam	Cat#AB416
anti-rabbit goat IgG AF488 (dilution 1:200)	Thermo Fisher Scientific	Cat#A-21206
anti-rabbit donkey IgG AF546 (dilution 1:200)	Thermo Fisher Scientific	Cat#A10040
anti-mouse donkey IgG AF546 (dilution 1:200)	Thermo Fisher Scientific	Cat#A10036
Anti-GLAST APC (dilution 1:100)	Miltenyi	Cat#130-098-803
Anti-GFAP Cy3 (dilution 1:200)	Sigma-Aldrich	Cat#C9205
Anti-S100β (rabbit) (dilution 1:200)	Abcam	Cat#AB52642

**Chemicals, peptides, and recombinant proteins**		

DMEM/F-12 + Glutamax	Gibco, Thermo Fisher Scientific	Cat#31331028
Neurobasal medium	Gibco, Thermo Fisher Scientific	Cat#21103-049
N2 supplement	Gibco, Thermo Fisher Scientific	Cat#17502001
B27 supplement without vitamin A	Gibco, Thermo Fisher Scientific	Cat#12587001
Laminin	Sigma-Aldrich	Cat#L-2020-1MG
Noggin	PeproTech	Cat#120-10C-20μG
SB431542	Tocris	Cat#1614
Human FGF-basic	PeproTech	Cat#100-18B
Human EGF premium grade	Miltenyi	Cat#130-097-750
StemMACS Y27632	Miltenyi	Cat#130-106-538
Penicillin/Streptomycin	Bioconcept	Cat#4-01F00-H
Human LIF	PeproTech	Cat#300-05
Human CNTF	PeproTech	Cat#450-13
TrypLE™ Express Enzyme (1**×**), phenol red	Gibco, Thermo Fisher Scientific	Cat#12605036
Matrigel	Corning	Cat#354277
Poly-L-ornithine solution 0.01%	Sigma-Aldrich	Cat#P4957-50ML
StemMACS™ iPS-Brew XF, human	Miltenyi	Cat#130-104-368
TeSR™-E8™	Stem Cell Technologies	Cat#05990

**Experimental models: Cell lines**		

hiPSC line HC1-C14 derived from a healthy donor	Perriot et al. 2018	N/A
hiPSC line HC2-C51 derived from a healthy donor	Perriot et al. 2018	N/A
hiPSC line HC2-C53 derived from a healthy donor	Perriot et al. 2018	N/A
hiPSC line HC3-C56 derived from a healthy donor	Perriot et al. 2018	N/A
hiPSC line HC3-C59 derived from a healthy donor	Perriot et al. 2018	N/A
hiPSC line MS1-C05 derived from a multiple sclerosis patient	Perriot et al. 2018	N/A
hiPSC line MS1-C24 derived from a multiple sclerosis patient	Perriot et al. 2018	N/A
hiPSC line MS2-C07 derived from a multiple sclerosis patient	Perriot et al. 2018	N/A
hiPSC line MS2-C12 derived from a multiple sclerosis patient	Perriot et al. 2018	N/A
hiPSC line MS3-C08 derived from a multiple sclerosis patient	Perriot et al. 2018	N/A
hiPSC line MS3-C13 derived from a multiple sclerosis patient	Perriot et al. 2018	N/A
hiPSC line MS4-C03 derived from a multiple sclerosis patient	Perriot et al. 2018	N/A
hiPSC line MS4-C07 derived from a multiple sclerosis patient	Perriot et al. 2018	N/A

**Other**		

Mr. Frosty	Sigma-Aldrich	Cat#C1562-1EA

## Materials and equipment

**Table:** Neural induction medium

Reagent	Final concentration	Amount
DMEM/F-12 + Glutamax		485mL
N2 supplement	1**×**	5mL
B27 supplement without vitamin A	1**×**	10mL
Noggin	500 ng/mL	250μg
SB431542	20 μM	10μmol
FGF-2	4 ng/mL	2μg
Laminin	2 μg/mL	1mg

**Table:** NPC expansion medium

Reagent	Final concentration		Amount	
DMEM/F-12 + Glutamax		485mL		
N2 supplement	1**×**	5mL		
B27 supplement without vitamin A	1**×**	10mL		
FGF-2	10 ng/mL	5μg		
EGF	10 ng/mL	5μg

**Table:** Astrocyte induction medium

Reagent	Final concentration		Amount	
DMEM/F-12 + Glutamax		485mL		
N2 supplement	1**×**	5mL		
B27 supplement without vitamin A	1**×**	10mL		
EGF	10 ng/mL	5μg		
LIF	10 ng/mL	5μg

**Table:** Astrocyte mediumS

Reagent	Final concentration		Amount	
DMEM/F-12 + Glutamax		490mL		
B27 supplement without vitamin A	1**×**	10mL		

## Step-by-step method details

### Differentiation of human iPSCs into neural precursor cells

**Timing: 4–5 weeks**

This section provides the detailed steps for obtaining neural precursor cells (NPCs) from hiPSCs. The first stage is based on the well established dual inhibition of SMAD signaling (Chambers et al., 2009), using SB431542 together with Noggin for concomitant inhibition of TGFβ and BMP signaling. Other BMP inhibitors such as LDN193189 or dorsomorphin can be used instead of noggin. The second stage describes the amplification of NPCs leveraging FGF2 and EGF signaling (Boissart et al., 2013).

***Note:*** Ensure of the hiPSC quality before starting the protocol. Human iPSCs should be at 70%–80% confluence and colonies to present an homogeneous aspect without differentiated cells.

1. On day 0, remove culture medium and add 1 mL of neural base medium to remove debris and cells in suspension from the dish.
2. Remove medium and add 1 mL of Neural induction medium + Rock inhibitor Y27632 (10μM).
3. With a cell scraper, draw successive vertical lines in the 35 mm dish to cut iPSC colonies as shown below. Then rotate the dish by 90° and redraw the same pattern to obtain squares. Rotate the dish once more by 45° and redraw the same pattern. See Figure 1.

**Figure 1:**
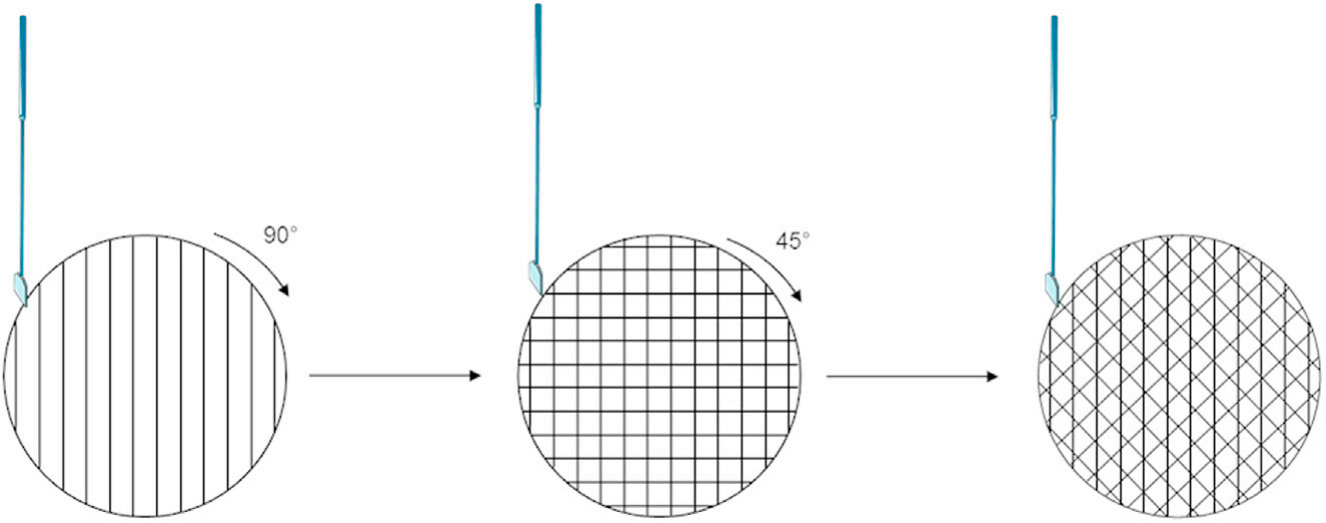
Schematic to generate hiPSC clumps for spheroid formation
4. Use the cell scraper to lift the clumps of iPSCs by gently scraping the bottom of the dish.
5. Gently transfer the entire clump suspension into a well of low-binding 6-well plate and add 1 mL of Neural induction medium + Rock inhibitor Y27632 (10μM). Incubate at 37°C for 4–6hrs until the clumps have formed spheroids.
6. Transfer the entire spheroid suspension to a new 35 mm dishes coated with poly-L-ornithine/Laminin (PO/L). Add 8μL/mL of Laminin to the medium for spheroid attachment. Incubate at 37°C. The next day, most of the spheroids should have adhered to the plate. Troubleshooting 1

**CRITICAL:** We found this way of forming spheroids to give the best results in term of purity of NPCs obtained. However, because it may be technically cumbersome to perform this technique without training, alternative methods can be used at this step: either spheroid formation using Aggrewell plates (STEMCELL Technologies), or induction after single cell hiPSC passaging such as described by Chambers et al.

**Figure 2:**
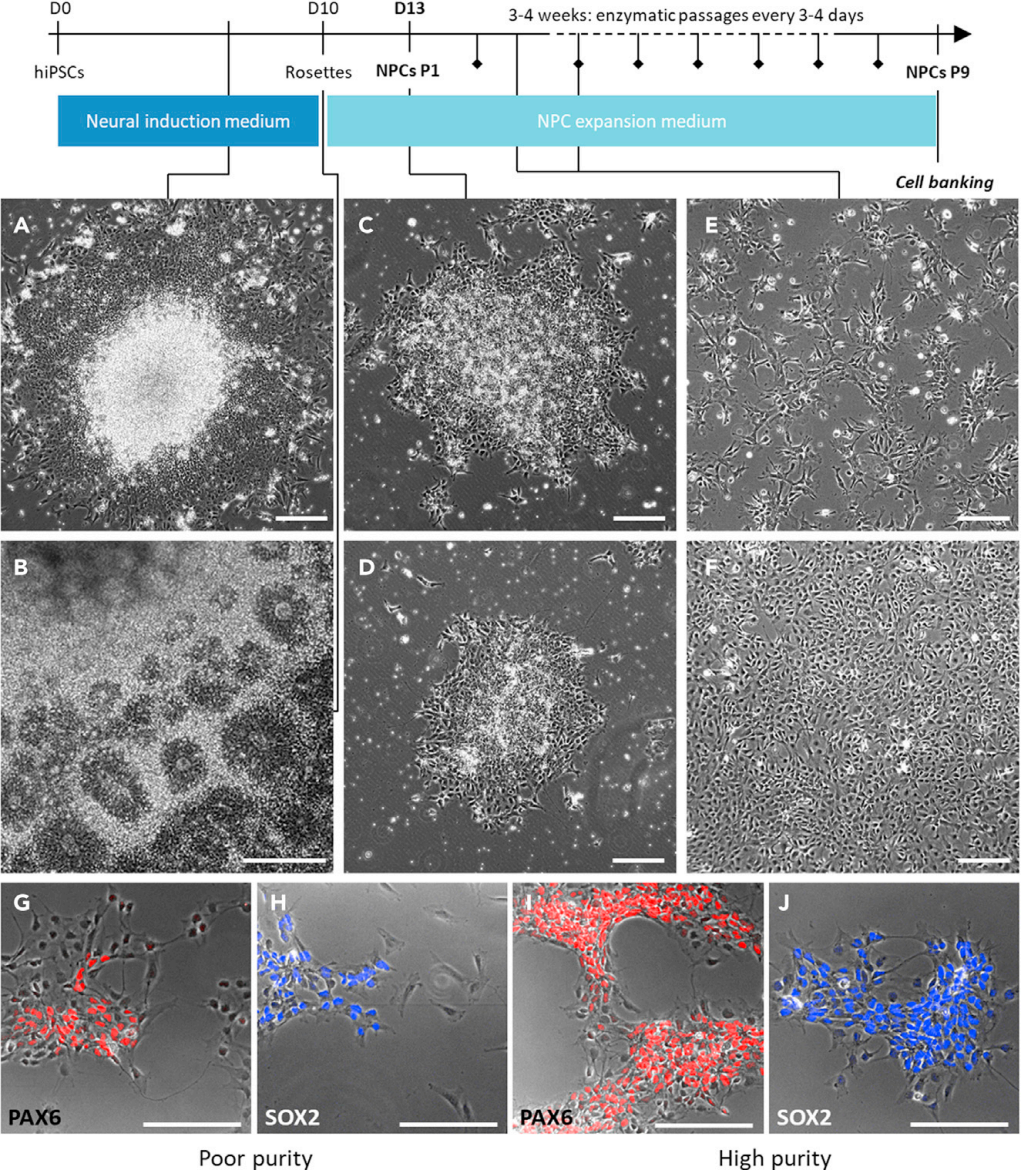
Overview of the protocol generating hiPSC-derived NPCs This timeline recapitulates the main steps to generate NPCs from hiPSCs with representative pictures of the expected cellular morphology at each intermediate: rosette appearance (A and B), clumps one day after rosette harvesting (C and D), NPCs 2 days after splitting (E) and NPCs at 100% confluent before splitting (F). Pictures (G and H) show an NPC population with low purity as assessed by the number of cells negative for PAX6 (G) and SOX2 (H). Conversely, pictures (I and J) show highly enriched NPCs with almost all cells expressing PAX6 (I) and SOX2 (J). The protocol can be paused and cells frozen for further use at P9 after NPC amplification. In the timeline, lozenges represent when an enzymatic passage should be performed (see step 13 for the procedure). Scale bar: 200 μm.
8. On day 10, remove neural induction medium and add 2 mL of NPC expansion medium.

***Note:*** Rosettes should be numerous by Day 10, either as monolayers or as multiple layers (Figure 2B).

7. Change medium every other day. Check cell morphology every time for rosette appearance (Figure 2A).

9. On day 13, change medium as of Day 10. Select areas with high density of rosettes, cut them in squares with a needle, and lift them individually with a 200 μL micropipette. Troubleshooting 2

**CRITICAL:** We recommend replating lifted rosettes on the same surface as previously cultured. Overall and after all the amplification steps, one 35mm dish of rosettes generates hundreds of millions of NPCs at the cell banking stage, pause point after step 15.

**CRITICAL:** The method described above generates a population of NPCs with the highest degree of purity (>95%). However, this method is time consuming and requires having a microscope under the laminar flow for optimal performance. Alternatively, rosettes can be harvested using the STEMdiff™ Neural Rosette Selection Reagent (STEMCELL Technologies). Please refer to manufacturer’s instructions: link. Keep in mind that the efficacy of this reagent is hiPSC line dependent. A third possibility, in case of very high homogeneity in the culture dish, is to perform an enzymatic passage to harvest all cells at once and then to purify them as described in the troubleshooting 2 section.

10. Transfer the clumps from the 35 mm dish to a new PO/L-coated 35 mm dish. From then, the cells are called NPCs and are at passage 1. Add Rock inhibitor Y27632 (10μM) to the medium (Figures 2C and 2D).
11. On day 14, change NPC expansion medium and incubate the cells at 37°C.
12. Change medium every other day and passage them after 6–7 days or at 80%–100% confluence if earlier. At this stage, we expect a yield of about 70′000 to 100′000 cells/cm^2^ so one 35 mm dish should yield enough cells for one to two T25 flasks.
13. Split the cells using TrypLE:
  a. Remove the culture medium and add 1 mL of TrypLE express per 35 mm dish (add 5 mL for a T75 flask). Incubate the cells at 37°C minutes for 10 min.
  b. Harvest the cells in flacon tubes and add twice the volume of DMEM/F-12. Centrifuge 5 min at 300 *g*.
  c. Resuspend the cell pellet in culture medium and count the cells.
14. Seed the cells in NPC expansion medium on matrigel-coated flasks (scale up flask size as needed) at 50′000 cells/cm2. Incubate at 37°C. NPCs are now at P2. Figures 2E and 2F show representative pictures of NPC morphology during amplification.

***Note:*** Cellular identity of NPCs can be assessed from this point: >80% of cells obtained should express PAX6 and SOX2 (Figures 2G–2J).

15. Change medium every other day. Split NPCs when confluent. Cell growth should allow splitting every 3–4 days. Each splitting increases the passage number by 1.

***Note:*** Cells can be amplified for a total of 8 passages and banked at P9 at 5 million cells per vial.

**Pause point:** Pausing at this step allows generating important batches of NPCs with high survival after thawing (>70%) to directly start astrocyte differentiation from this step (troubleshooting 3). See additional procedures at the end of the protocol for cell freezing. See Figure 2.

### Differentiation of hiPSC-derived NPCs into astrocytes

**Timing: 6 weeks**

This section describes the steps to differentiate previously obtained NPCs into astrocytes. It relies on activation of the JAK/STAT pathway by concomitant exposure of NPCs to EGF and LIF in order to accelerate the glial switch (Bonni et al., 1997) in absence of serum. LIF is instrumental to activate the JAK/STAT pathway while EGF inhibits premature differentiation of NPCs into neurons. Maturation of astrocytes is obtained by exposure to CNTF. This step can be performed after freezing/thawing of NPCs but has to be conducted to the end without pause once started. Thus, counting of days of differentiation restart at 0.

***Optional:*** In case a pause has been made between steps 15 and 16, cells need to be thawed to perform astrocyte differentiation. See additional procedures at the end of the protocol for cell thawing.

**Figure 3:**
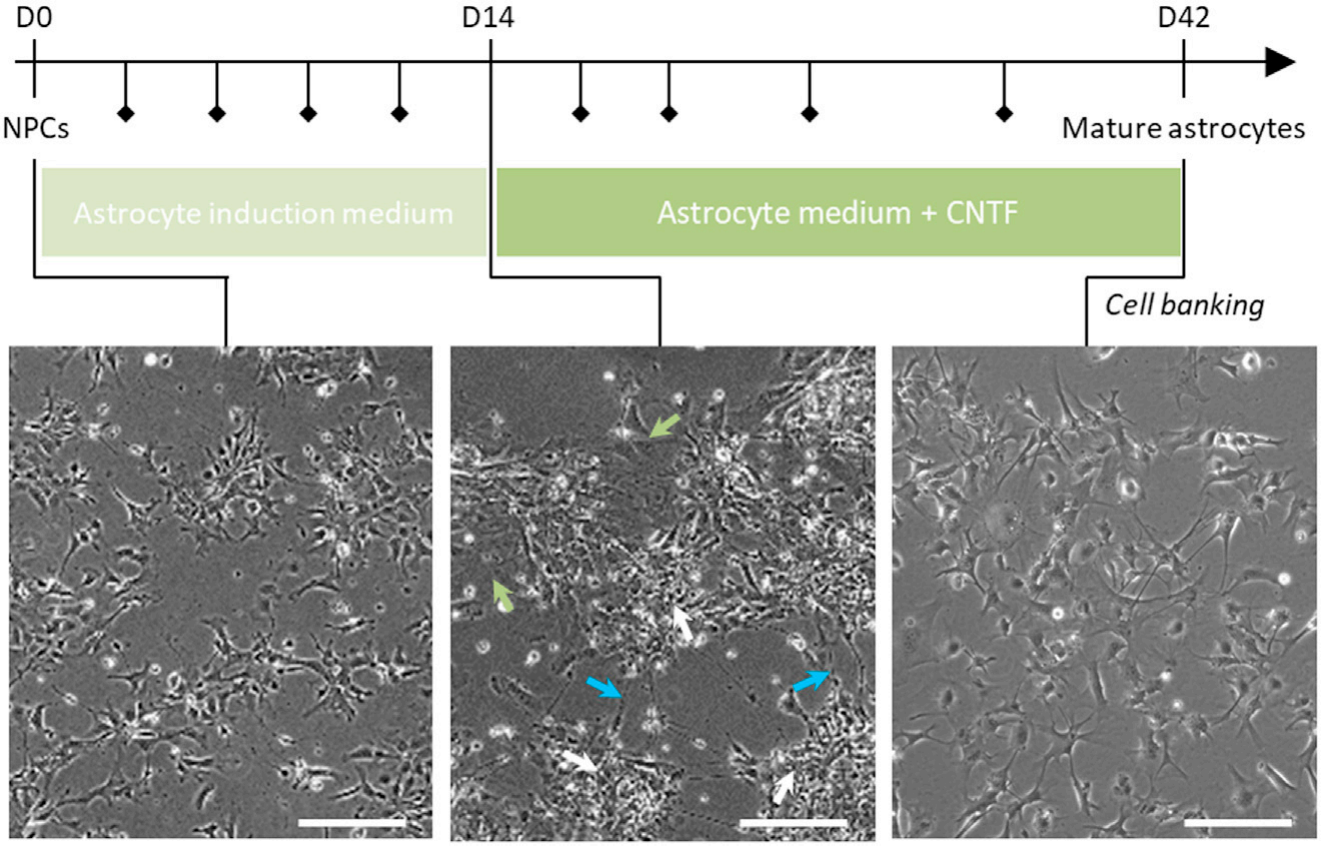
Overview of the protocol generating hiPSC-derived astrocytes from NPCs This timeline recapitulates the main steps to generate astrocytes from hiPSC-derived NPCs with representative pictures of the expected cellular morphology at each intermediate: thawed NPCs, differentiating cells and mature astrocytes. Cells can be banked at the end of the protocol after astrocyte maturation. On the central panel, white arrows indicate NPC niches, blue arrows indicate differentiating neurons and green arrows indicate emerging astrocytes. In the timeline, lozenges approximately represent the frequency of enzymatic passages (see steps 13 to 15 for the procedure). Scale bar: 200 μm.
17. Change medium every other day for 14 days. Slit cells with TrypLE as described on Step 13 each time they reach confluence during this step (about every 3–4 days).

***Note:*** This step serves to trigger the glial switch in NPCs. It thus decreases the potential of NPCs to generate neurons and changes the epigenetic landscape of the cells to allow astrocyte generation. There is no need for massive amplification of the cells at this stage as the cells retain a certain proliferation potential until D21.

16. At D0 of astrocyte differentiation, change NPC culture medium to Astrocyte induction medium (Figure 3, left panel).

18. At D14, switch medium to Astrocyte medium + CNTF (20 ng/mL). Cells start to differentiate, and neurons and astrocytes can be seen in the culture (Figure 3, central panel).
19. From D14 to D28, change medium every other day and passage cells when at confluence (do not passage them if confluence is <90%–100%, however cells can be allowed to overgrow). Seed cells at 40′000 cells/cm^2^ onto matrigel-coated flasks and incubate at 37°C. Refer to troubleshooting 3 for evaluating how many flasks are needed depending on the expected yield of astrocytes.

**CRITICAL:** Proliferation will decrease as NPCs differentiate. The extent of this decrease is dependent upon each iPSC line. Neurons may appear in the culture concomitantly to astrocytes. Overall, neurons will die at each splitting to let only astrocytes in culture at the end of the protocol. Between D14 and D21, cells should mostly have an NPC morphology with some neurons. Between D21 and D28, a mix of NPCs, neurons and immature astrocytes should be present in the culture. From D28, astrocytes should start to represent most of the cells in culture. Some neurons and NPCS may remain until D35 but should be scarce. Troubleshooting 4.

20. From D29 to D42, change medium once every three to four days and plate cells at 20′000 to 30′000 cells/cm^2^ passaging them as described in step 13.

***Note:*** As astrocytes mature, they become bigger and have to be plated at a lower density to allow proliferation and obtain a good yield.

21. After D42, cells are mature and can be used for downstream experiments (Figure 3, right panel). Culture cells in Astrocyte medium without CNTF from that day onward. Astrocytes retain their phenotype until at least D70 but should be passaged as rarely as possible.

***Optional:*** Cells can be frozen in Astrocyte medium + 10% DMSO at this stage. Cells retain their phenotype and functionality after thawing. Viability after thawing is usually between 50% to 70%. Troubleshooting 5.

Additional procedures:

22. Cell freezing
  a. Detach the cells from the plate with TrypLE and harvest them.
  b. Count them and centrifuge 5 min at 300 *g*.
  c. Resuspend the cells at 5 million/mL in Astrocyte medium supplemented with DMSO 10%.
  d. Distribute 1 mL per cryovial and store at −80°C in a Mr Frosty^TM^ container. Transfer the cells in liquid nitrogen after 3 days for long-term storage.
23. Thawing procedure:
  a. Prepare a falcon tube containing 9 mL of astrocyte medium per vial to be thawed.
  b. Incubate one vial of NPCs in a water bath at 37°C for 1 min.
  c. Transfer the content of the vial into the falcon tube with astrocyte medium.
  d. Centrifuge for 5 min at 300 *g*.
  e. Resuspend the cell pellet in 10 mL of cell culture medium supplemented with Y27632 (10 μM).
  f. Plate the cells in one matrigel-coated T75 flask.
  g. Change the medium after 24hrs with culture medium to remove Y27632.

## Expected outcomes

**Figure 4:**
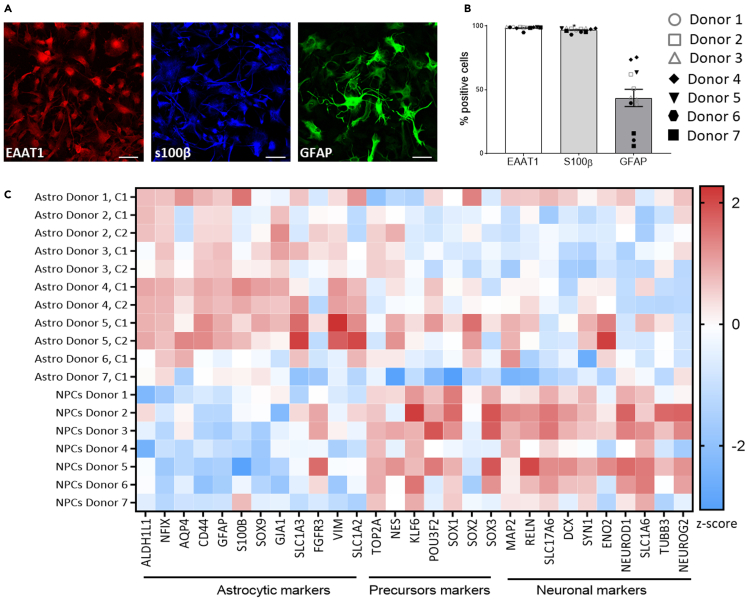
Characterization of hiPSC-derived astrocytes Astrocytes were derived from seven donors according to the present protocol. Two hiPSC lines were derived for four donors and one hiPSC line for three donors. Astrocytes express canonical astrocytic markers as monitored by immunofluorescence staining for EAAT1 (red), S100β (blue) and GFAP (green) (A). More than 95% of the cells in culture co-express both EAAT1 and S100β while GFAP is expressed by a variable proportion of the cells (B). As compared to parental NPCs, astrocytes display increased expression of typical astrocyte markers associated with a downregulation of NPCs and neuronal markers (C). Scale bar: 100 μm. SB: SB431542, FGF2: fibroblast growth factor 2, EGF: epithelium growth factor, LIF: leukemia inhibitory factor, CNTF: ciliary neurotrophic factor. Adapted from (Perriot et al., 2018) according to the CC BY license 4.0.

The protocol described here allows obtaining highly enriched culture composed of more than 95% of human astrocytes (Table 1) as displayed in Figure 4. The total yield of astrocytes generated depends only on the amplification strategy decided by the operator (Table 2). One single operator can handle the generation of more than 100 million astrocytes per differentiation providing that the surface of cell culture is regularly increased to match the number of cells generated. See troubleshooting 3 for more details.

**Table 1:** Cell population generated during this protocol and related markers

Stage	Markers	Purity
hiPSCs	Tra-1-60, OCT4	>95%
NPCS	PAX6, SOX2, PSA-NCAM	>90%
Astrocytes	EAAT1, S100β	>95%

**Table 2:** Yields according to differentiation stages

Stage	Starting cells	Intermediates	End product
NPCS	hiPSCs, 1**×** 35 mm dish	Rosettes, 1**×** 35 mm dish	>100 million NPCs
Astrocytes	5 million NPCs (fresh or thawed)	N/A	>100 million astrocytes

Functional characterization of the astrocytes is strongly advised. Please refer to the supplemental information from Perriot et al., (2018) for all detailed methods.

## Limitations

The protocol detailed here reproducibly generates a highly enriched population of functional astrocytes without the use of serum. This method presents several advantages such as the generation of astrocytes with a resting and less altered phenotype as compared to astrocytes cultured in serum, yet removing serum from culture medium renders more difficult the glial switch in NPCs. As a result, the final yield and timing of astrocytes may vary depending on the hiPSC line used. First, some hiPSC lines generate NPCs demonstrating resistance to acquire gliogenic potential. As a consequence, the optimal duration of priming with EGF and LIF should be empirically determined (elongated) if the timing described here fails to generate high yields of astrocytes. Second, mature astrocytes are big cells and the number of cells generated per cm^2^ is often low as compared to other cell types. This characteristic renders cumbersome the generation of important batches of cells and requires to perform cell culture on a large surface (>1000cm^2^).

## Troubleshooting

### Problem 1

Low adherence of spheroids

The day after plating at step 6, most spheroids should have adhered to the dish. In some occasion, many spheroids may remain in suspension.

### Potential solution

Harvest the spheroids in a 15-mL falcon tube. Let the clumps to sediment at the bottom of the tube. Carefully remove the supernatant in the tube and add 2 mL of Neural induction medium with Laminin at 8 μg/mL. Replate the clumps in a new PO/L-coated 35 mm dish. Then continue the protocol from step 7.

### Problem 2

Presence of non-astrocytic cells

At the end of the protocol, more than 95% off cells should be astrocytes (double positive for EAAT1 and S100β together with the corresponding morphology). If it is not the case, it is most likely due to contaminant cells at the NPCs stage, i.e., some cells composing the population generated at step 9 are not NPCs.

### Potential solution

We recommend to attentively follow the instructions at step 9 and to take the maximal care of harvesting only neural rosettes as cells outside of these rosettes may be of other lineages than NPCs (neural crest cells for example). The population resulting from rosette selection should be negative for CD271 (<10%) and positive for CD56 (>90%). If the rosettes are not clearly identifiable, it is mostly due to the generation of hiPSC clumps that would be too small at step 4. An alternative solution to enrich the culture in NPCs, in case selection of rosettes alone would not be possible, is to purify the cells based on surface marker expression. We suggest using flow cytometry or magnetic-activated sorting based on PSA-NCAM marker right after step 9.

### Problem 3

Low NPC survival after thawing

After thawing, survival of NPCs should reach around 70%. In rare occasions, survival may be lower and prevent correct execution of the differentiation (step 16).

### Potential solution

Poor survival arises from two different factors: poor execution of the freezing/thawing procedure or poor cell quality at freezing.

One key aspect of optimal freezing and thawing is a rapid execution. Cells should remain in liquid freezing medium the least time possible, both before freezing and after thawing. We recommend to have all reagents, media and falcon tubes ready when starting the procedures. Transfer to the −80°C freezer should be done without waiting once cells are resuspended in the freezing medium during freezing. Use of containers (MrFrosty^TM^) allowing a progressive decrease of temperature greatly increases cell survival. For thawing, the freezing medium should be diluted at least 10 times as soon as possible after thawing of the vial.

Poor cell quality at freezing is independent on the freezing procedure and should be avoided at all cost as such cells will not be usable for further differentiation and the protocol should be restarted from the beginning. It may happen if cells are over confluent on the day of freezing thus we recommend performing cell banking at 80%–90% of confluence to ensure that the cells are as healthy as possible.

### Problem 4

Low yield of astrocytes

Conducted correctly, the protocol can yield more than 100 million cells. Lower yields can be intentional by reducing the surface of culture. Indeed, generating that many cells is costly in terms of hands-on time and reagents. Yet unwanted low yield may happen for two main reasons: low gliogenic potential of NPCs after step 17 or poor amplification strategy.

### Potential solution

In case of low gliogenic potential, NPCs will mostly differentiate into neurons, which can be observed already by D21. The switch from neurogenic potential to gliogenic potential is variable depending on each hiPSC line. The timing proposed here should work for most lines but could be too short for some. In case of too abundant neuronal differentiation, we recommend to extend the culture with the Astrocyte Induction medium by one to two weeks. Glial switch can be monitored by assessing the expression of transcription factors such as NeuroD2 and NeuroG2 (downregulation in gliogenic NPCs) and SOX9 and NFIA (upregulation in gliogenic NPCs).

In case of low yield due to a poor amplification strategy, the operator should keep in mind that astrocytes should be plated at low density as they become mature thus requiring an important cell culture surface. About 30′000 to 40′000 cells can be obtain per cm^2^. As cells proliferate less with maturation, the operator should anticipate this effect and perform immature astrocyte amplification between D14 and D28. A total culture surface of around 2000–2500cm^2^ at D35 should allow generating about 100 million astrocytes if NPCs have developed a good gliogenic potential between D0 and D14 (see point above). See Table 3 below for seeding density at each stage and suggested cell culture surface.

**Table 3:** Seeding density and suggested cell culture surface

Stage	Seeding density	Cell culture surface
NPCs (expansion stage)	50′000 cells/cm^2^	From 1**×**T25 (25cm^2^) at P1 to 4**×**T150 at P8 (600cm^2^)
Astrocyte induction stage, D0 to D14	50′000 cells/cm^2^	2**×**T150 at D14 (300 cm^2^)
Astrocyte maturation stage, D14 to 28	40′000 cells/cm^2^	Up to 6-8**×**T150 at D28 (900–1200cm^2^)
Astrocyte maturation stage, D29 to 42	20′000–30′000 cells/cm^2^	Up to 12-16**×**T150 at D42 (1800–2400cm^2^)

### Problem 5

Low astrocyte survival after thawing

After thawing, survival of astrocytes should reach 50%–70% of frozen cells. Lower cell survival results from non-optimal cell handling and freezing (step 21).

### Potential solution

As mentioned in troubleshooting 3, freezing and thawing procedures must be executed quickly. Please refer to troubleshooting 3 for the aspects of the method. In addition, cellular density at freezing impacts on cell survival at thawing. We recommend freezing astrocytes at 3–5 million cells/mL. Lower density will result in low cell survival. Higher density (tested up to 7 million/mL) does not result in higher survival.

## Resource availability

### Lead contact

Further information and requests for resources should be directed to the lead contact, Renaud Du Pasquier, renaud.du-pasquier@chuv.ch.

### Materials availability

This study did not generate any unique reagents.

## Data Availability

This study did not generate any unique data sets or code.
